# Changes in objectively measured sleep among internationally adopted children in 1-year follow-up during the first years in new families

**DOI:** 10.3389/fped.2022.948010

**Published:** 2022-09-09

**Authors:** Anna-Riitta Heikkilä, Helena Lapinleimu, Irina Virtanen, Hanni Rönnlund, Hanna Raaska, Marko Elovainio

**Affiliations:** ^1^Department of Pediatrics, University of Helsinki, Helsinki, Finland; ^2^Department of Pediatrics, Helsinki University Hospital, Helsinki, Finland; ^3^Department of Paediatrics and Adolescent Medicine, University of Turku, Turku, Finland; ^4^Department of Paediatrics and Adolescent Medicine, Turku University Hospital, Turku, Finland; ^5^Department of Clinical Neurophysiology, Turku University Hospital, Turku, Finland; ^6^Department of Clinical Neurophysiology, University of Turku, Turku, Finland; ^7^Kaarina Health Center, Kaarina, Finland; ^8^Department of Child Psychiatry, Helsinki University Hospital, Helsinki, Finland; ^9^Department of Psychology and Logopedics, University of Helsinki, Helsinki, Finland; ^10^Department of Health Services Research, National Institute for Health and Welfare, Helsinki, Finland

**Keywords:** sleep, sleep of children, internationally adopted children, actigraphy, FinAdo

## Abstract

**Background:**

Psychosocial risks and environmental changes experienced by internationally adopted children may predict sleep problems, which are incidentally among the main concerns of adoptive parents. Several questionnaire studies have found sleep of internationally adopted children to be problematic, but none of those used an objective measure in a controlled study.

**Objective:**

To determine whether the objectively recorded sleep of internationally adopted children is worse than their controls who are living with their biological parents.

**Methods:**

To this case-control part of the Finnish Adoption Study, we recruited children who were adopted internationally to Finland between October 2012 and December 2016. Simultaneously, control children were recruited from 16 daycare centers. To assess sleep in children, actigraphy recordings were made twice, 1 year apart, between December 2013 and April 2018. In the adopted group, the first assessment took place 10 months after they had arrived in their families. The associations between adoption status and sleep parameters were analyzed using linear mixed modeling and adjusted for multiple potential confounders, including child age.

**Results:**

Seventy-eight internationally adopted children (boys 64%) aged 1–7 years and 99 controls (boys 53%) aged 2–6 years attended the first sleep recording. The recordings showed that the internationally adopted children slept longer (B = 0.48, 95% CI 0.23–0.73, *P* < 0.001) than the controls. There were no significant differences in sleep fragmentation or sleep efficiency between the groups. During the 1-year follow-up, the sleep patterns of the adopted children approached those of the controls.

**Conclusions:**

The internationally adopted children spent more time in bed and slept more than their control children in both recordings. However, their sleep patterns were not very different from those of their peers and the differences appeared to vanish during the first years in their new family.

## Introduction

About a quarter of parents with small children report sleep difficulties in their children (1–5). Even up to 50% of the parents of internationally adopted children report sleep problems in their children, including bedtime resistance, sleep onset delay, sleep anxiety, restless sleep, parasomnias, nightmares, and frequent nocturnal awakenings, during the first years in the family (6–13). In a recent study, almost a third of adolescents adopted in childhood had insomnia and this proportion was significantly larger than in their non-adopted controls (14). Pre-adoptive stress, lack of parental care, deprived living conditions in institutions, and major changes during the adoption transition have been suggested as risk factors for sleep disturbances in adopted children (6, 15–17).

Previous evidence on sleep problems among internationally adopted children is mixed. In some studies, internationally adopted children have clearly had more sleep problems than other children (8–10), while other studies show that their sleep was not worse than that of children in the general population (7, 13). Earlier studies on sleep in internationally adopted children have employed parental or self-reports and various questionnaires to define types of sleep disturbances (7, 9, 10, 13, 14), and only a few of them have used standardized sleep surveys (9, 10, 13). Some studies have shown that parents may overestimate their child's sleep disturbances in self-reported sleep questionnaires compared to objective markers (18–24). Only two of the previous studies on sleep in internationally adopted children have defined sleep patterns, such as total sleep time, sleep latency, restlessness of sleep, and sleep efficiency. However, neither utilized objective methods, were based on questionnaires, and sleep patterns were calculated based on parental and self-reports (10, 14).

We aimed to use actigraphy to investigate the objective patterns and development of sleep among internationally adopted children compared to controls living with their biological parents. We hypothesized that internationally adopted children sleep more poorly than their controls, they have difficulties initiating sleep, and their sleep is more fragmented and inefficient.

## Materials and methods

### Participants

The Finnish Adoption (FinAdo 2) Study is an ongoing follow-up study on the wellbeing of internationally adopted children in Finland. The data used in this study are part of a clinical follow-up study conducted between December 2013 and April 2018, which included 177 participants (78 adopted children and 99 controls at the first assessment, Table 1) and information on sleep variables (measured using actigraphy) from at least one assessment. No children attended the second phase without attending the first phase of the study. On the second assessment, reliable actigraphy data were obtained from 66 adopted children (85%) and 77 controls (80%).

**Table 1:** Participant characteristics based on the adoption status.

		**Adopted**		
		**Yes**	**No**	***P*** **-value for difference**
**Children**				
Age at Phase 1 (years)	Mean (SD)	3.6 (1.8)	4.8 (1.3)	<0.001
Gender, *n*	Girl (%)	28 (36)	47 (47)	0.16
	Boy (%)	50 (64)	52 (53)	
Number of siblings, *n*	Mean (SD)	0.5 (0.9)	0.8 (1.1)	0.11
Basic health status, *n*	Healthy (%)	67 (88)	94 (95)	0.17
	Special needs^*^ (%)	9 (12)	5 (5)	
Room-sharing with parents, *n*	Yes (%)	35 (47)	26 (26)	0.007
	No (%)	39 (53)	73 (74)	
**Mothers**				
Mother's age	Mean (SD)	41.0 (5.2)	36.2 (4.9)	<0.001
Mother's depressive symptoms	Mean (SD)	1.9 (0.4)	2.0 (0.4)	0.41
Mother's sleep problems	Mean (SD)	2.5 (0.8)	2.4 (1.0)	0.32
Mother's education	Primary school (%)	0 (0)	4 (4)	0.31
	High/vocational school (%)	7 (15)	18 (18)	
	University (%)	40 (85)	77 (78)	
Marital status, *n*	Married or cohabiting (%)	39 (87)	79 (80)	0.45
	Unmarried or single parent (%)	6 (13)	20 (20)	
**Sleep variables**				
Number of nights registered	4 (%)	0 (0)	2 (2)	0.42
	5 (%)	1 (1)	0 (0)	
	6 (%)	7 (9)	9 (9)	
	7 (%)	70 (90)	88 (89)	
Total sleep time in 24 h	Mean (SD)	9.2 (0.8)	8.6 (0.5)	<0.001
Total nocturnal sleep hours	Mean (SD)	8.5 (0.6)	8.2 (0.5)	<0.001
Sleep fragmentation	Mean (SD)	40.9 (8.7)	37.6 (7.7)	0.009
Nocturnal sleep fragmentation	Mean (SD)	41.9 (9.5)	38.1 (8.0)	0.005
Sleep latency	Mean (SD)	0.3 (0.2)	0.4 (0.2)	0.11
Nocturnal sleep latency	Mean (SD)	0.4 (0.2)	0.4 (0.2)	0.74
Time in bed (hours)	Mean (SD)	11.8 (1.2)	11.0 (0.7)	<0.001
Nocturnal time in bed (hours)	Mean (SD)	10.9 (0.7)	10.4 (0.6)	<0.001
Sleep efficiency	Mean (SD)	77.8 (5.4)	78.2 (3.9)	0.54
Sleep efficiency at night	Mean (SD)	78.2 (5.5)	78.9 (4.0)	0.33
Cosine peak	Mean (SD)	14.5 (0.9)	14.2 (0.8)	0.03
Season, Phase 1, *n* (%)	Spring	25 (32)	35 (50)	<0.001
	Summer	4 (5)	14 (20)	
	Autumn	17 (22)	2 (3)	
	Winter	33 (41)	19 (27)	
Season, Phase 2, *n* (%)	Spring	29 (44)	29 (42)	0.27
	Summer	3 (5)	3 (4)	
	Autumn	8 (12)	17 (25)	
	Winter	26 (39)	20 (29)	

* Structural defect, global development delay, or other disability.

Statistical significances are based on chi-square tests for the categorical explanatory variables and the F-test (ANOVA) for the continuous variable.

We recruited internationally adopted children who arrived in southern Finland below the age of 7 between October 2012 and December 2016. Invitation letters were sent through three authorized adoption service providers at the time when the families were still in the adoption process and waiting for their children. At the first actigraph measurement, the mean age of the adopted children was 3.6 years (ranging from 1 to 7 years, SD 1.8 years). They had moved to Finland, on average, 0.8 years (SD 0.5) earlier through international adoption. Of them, 49% were from Asia, 37% from Africa, 6% from South America, and 8% from Eastern Europe. Most of these children were adopted from orphanages (77%) and only 4% were adopted from foster homes. Of them, 19% had more than two pre-adoptive placements.

The children in the control group lived with their biological parents. We recruited them from 16 daycare centers in the cities of Turku and Kaarina in Southwest Finland (19), and their age ranged from 2 to 6 years (mean 4.8, SD 1.3). Two children from this group were excluded because they were also adopted internationally a few years earlier.

The children were divided into two health categories: healthy children or children with special needs (25), such as structural defects, global developmental delay, or other disabilities (Table 1). The main exclusion criteria were a long-term medication that affects sleep or a diagnosis of obstructive sleep apnea. Two adopted children from the original cohort were excluded from this part of the study. One adopted child and 8 children from the original control group were excluded after declining to wear an actigraph or because of an incomplete recording due to mostly technical reasons at the first recording. On the second phase, those children who did not provide reliable data at the first recording were excluded and one recording among the adopted children produced a blank result, i.e., the actigraphy bracelet had not measured the data. Adopted children with congenital structural defects that could affect their sleep, such as a cleft lip or palate (*n* = 6), were included in the study, but their sleep was also analyzed separately.

### Sleep measures

We assessed sleep characteristics between December 2013 and April 2018 twice (1 year apart) during two data collection phases (Phase 1 and Phase 2) using an actigraphy bracelet (GeneActiv Original; Activinsights Ltd., Kimbolton, Cambridgeshire, United Kingdom) (26), a reliable method that objectively evaluates sleep patterns in children (27, 28). The actigraph was worn on the child's non-dominant wrist for 7 days and was removed only during bath time and participation in contact sports. The parents and daycare personnel were advised to press the event button of the actigraph when the child went to bed and got up both at night and during the daytime naps. They were also guided to use sleep diaries to take a note of every bedtime and get-up time and every instance the bracelet was removed, followed by the reason.

The actigraph recorded physical activity and enabled calculated estimations of the sleep parameters used in this study: time in bed in hours and minutes, total sleep time in hours and minutes, sleep latency, sleep efficiency, fragmentation index, and cosine peak (28). Sleep latency was measured from bedtime to sleep start; sleep efficiency was the percentage of time spent asleep in bed; the fragmentation index measured nocturnal restlessness; and the cosine peak measures the timing of a person's most active period in 24 h (timing of peak daytime activity). A research assistant and an experienced clinical neurophysiologist (I.V.) analyzed the actigraph data and checked incomplete actigraph event button marks of bedtimes and get-up times from the sleep diaries (19, 28).

### Potential mediators and confounders

Potential confounders included adoption status, age, gender, number of siblings, cosine peak, daycare attendance, co-sleeping with parents, parents' age, parental education, parents' sleep problems, parents' depressive symptoms, and the season of the data collection point (Table 1). Parents' sleep problems were measured using the Jenkins Sleep Scale (29) (4 items, ranging from 1 = never to 5 = every night, Cronbach's α = 0.67 from the current sample) and parents' depressive symptoms using the 12-item version of the General Health Questionnaire (30) (range 1–4, Cronbach's α = 0.88. from the current sample) (31). Parents' sleeping problems, psychological distress, and co-sleeping were reported in both data collection phases (Table 1).

### Statistical analyses

The data were structured so that each participant contributed one to two observations to the dataset, depending on the number of study phases for which data for that participant were available. This study design allowed us to use all available data, and the method considered the non-independence of repeated measurements (person-observations) on the same individual. The data were analyzed using mixed modeling (random intercept and random slope multilevel linear regression) and the model uses all observations and considers that they are clustered per individual, even if some individuals do not have second recording (32).

We examined whether adoption status was associated with changes in sleep characteristics over the study phases and whether there were significant differences in the trajectories of the sleep characteristics in a follow-up between adopted children and controls (testing the phase^*^adoption status interactions). The analyses included only those confounders that were significantly different between the controls and adopted children at the baseline (Phase 1).

The data analysis was carried out with R programming software 4.0.3 and the Imer4- and sjPlot- packages (33, 34).

### Ethics

The Ethics Review Committee of the Hospital District of Southwest Finland approved this study. We obtained written informed consent from the participants' parents.

## Results

**Figure 1:**
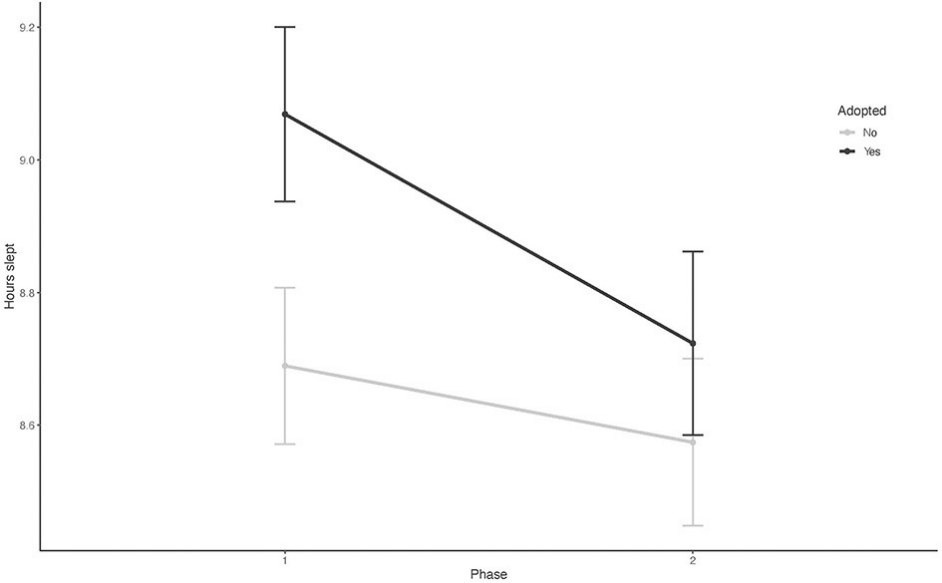
The mean trajectories for sleep duration in 24 h in the study population over the study recordings (Phase 1 and Phase 2).

**Figure 2:**
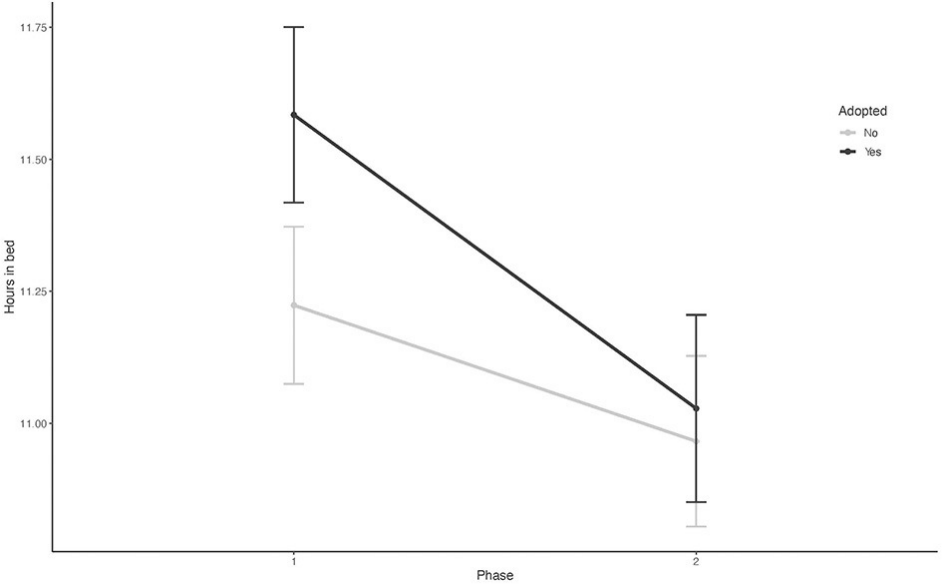
The mean time spent in bed in 24 h in both study groups over two study phases (Phase 1 and Phase 2).

The sample characteristics are shown in Table 1. Between the characteristics of the adopted children and the control children, there were no statistically significant differences between the groups in terms of gender, the number of siblings, or parental marital status. Of the 177 child participants, 66 adopted children (85%) and 77 controls (80%) provided information on sleep measures for both data collection phases. At the first recording, the adopted children stayed in bed 0.8 h longer and slept 0.6 h longer than the controls (Table 1). The repeated regression analyses over the whole recordings showed that the adopted children had longer times in bed in 24 h (B = 0.54, 95 % CI 0.23–0.85, *P* = 0.001) and longer total sleep times over 24 h (B = 0.48, 95% CI 0.23–0.73, *P* < 0.001) (Table 2; Figures 1, 2). Their sleep was more fragmented than that of the controls, but this difference disappeared with age adjustment, and their cosine peak was slightly later in Phase 1. In both groups, the children who shared a room with their parents slept less than those who slept in their own room (Table 3). There were no significant differences between neither the number of siblings nor in co-sleeping with siblings between the study groups, so that could not explain the differences in sleep patterns between adopted children and biological controls. The sleep of adopted children with a cleft lip or palate did not differ significantly from that of other adopted children. The background characteristics of the family differed significantly only in the age of the mothers between these two groups, with adoptive mothers being significantly older (Table 1).

**Table 2:** Associations between adoption status and changes in total 24 h sleep over the study phases.

	**Sleep time**		**Time in bed**		**Sleep latency**		**Sleep fragmentation**		**Sleep efficiency**	
**Predictor**	**Estimates**	***P*** **-value**	**Estimates**	***P*** **-value**	**Estimates**	***P*** **-value**	**Estimates**	***P*** **-value**	**Estimates**	***P*** **-value**
(Intercept)	9.31 (8.05–10.56)	**<0.001**	13.70 (12.02–15.38)	**<0.001**	0.02 (−0.40 to 0.43)	0.94	59.63 (43.45–75.81)	**<0.001**	66.51 (56.86–76.17)	**<0.001**
Phase (2)	−0.09 (−0.21 to 0.03)	0.14	−0.21 (−0.38 to −0.04)	**0.02**	0.04 (−0.01 to 0.09)	0.11	−2.72 (−4.24 to −1.19)	**0.001**	0.76 (−0.18 to 1.70)	0.11
Adopted (yes)	0.48 (0.23–0.73)	**<0.001**	0.54 (0.23–0.85)	**<0.001**	−0.03 (−0.10 to 0.04)	0.33	2.48 (−0.76 to 5.71)	0.13	0.55 (−1.20 to 2.53)	0.57
Age (years)	−0.16 (−0.23 to −0.09)	**<0.001**	−0.35 (−0.43 to −0.27)	**<0.001**	−0.01 (−0.03 to −0.00)	0.14	−1.69 (−2.56 to −0.82)	**<0.001**	1.02 (0.52–1.52)	**<0.001**
Gender (boy)	−0.05 (−0.24 to 0.13)	0.59	−0.01 (−0.24 to 0.21)	0.90	0.06 (0.01–0.10)	**0.02**	−0.74 (−3.15 to 1.68)	0.55	−0.51 (−1.89 to 0.87)	0.47
Cosine peak	0.01 (−0.06 to 0.09)	0.72	−0.05 (−0.15 to 0.05)	0.35	0.04 (0.02–0.07)	**0.001**	−0.79 (−1.73 to 0.15)	0.10	0.37 (−0.20 to 0.94)	0.21
Mother's age	−0.00 (−0.02 to 0.02)	0.84	−0.01 (−0.03 to 0.01)	0.45	−0.01 (−0.01 to −0.00)	**0.03**	−0.09 (−0.35 to 0.16)	0.47	0.06 (−0.08 to 0.21)	0.39
Room-sharing with parents	−0.22 (−0.38 to −0.05)	**0.009**	−0.20 (−0.41 to −0.01)	0.06	−0.06 (−0.11 to −0.01)	**0.02**	1.33 (−0.76 to 3.43)	0.21	−0.50 (−1.74 to 0.73)	0.42
Spring	−0.01 (−0.18 to 0.15)	0.88	0.01 (−0.22 to 0.24)	0.93	0.01 (−0.05 to 0.07)	0.81	−0.47 (−2.60 to 1.66)	0.67	−0.25 (−1.54 to 1.05)	0.71
Summer	0.07 (−0.16 to 0.29)	0.55	0.33 (0.01–0.64)	**0.04**	0.00 (−0.08 to 0.09)	0.92	2.30 (−0.55 to 5.16)	0.11	−1.71 (−3.46 to 0.03)	0.06
Winter	0.01 (−0.16 to 0.19)	0.87	0.13 (−0.10 to 0.37)	0.27	0.05 (−0.01 to 0.11)	0.10	0.78 (−1.42 to 2.98)	0.48	−0.64 (−1.97 to 0.69)	0.35
Phase ^*^ adoption status interaction	−0.26 (−0.43 to −0.09)	**0.003**	−0.38 (−0.63 to −0.12)	**0.004**	−0.02 (−0.09 to 0.05)	0.57	−0.09 (−2.29 to 2.11)	0.93	0.24 (−1.12 to 1.60)	0.73
**Random effects**										
σ^2^ (variance of model)	0.11		0.23		0.02		17.53		6.72	
τ_00_ (variance between individuals)	0.23_id_		0.30_id_		0.01_id_		39.34_id_		12.39_id_	
ICC (intraclass correlation)	0.68		0.56		0.27		0.69		0.65	
N	145_id_		145_id_		145_id_		145_id_		145_id_	
Observations	271		271		271		271		271	
Marginal R^2^/conditional R^2^	0.302/0.776		0.475/0.769		0.146/0.381		0.224/0.761		0.158/0.704	

The bold values are the values with statistical significance.

**Table 3:** Associations between adoption status and changes in nighttime sleep over the study phases.

	**Total nocturnal sleep time**		**Nocturnal time in bed**		**Nocturnal sleep latency**		**Nocturnal sleep fragmentation**		**Nocturnal sleep efficiency**	
**Predictor**	**Estimates**	***P*** **-value**	**Estimates**	***P*** **-value**	**Estimates**	***P*** **-value**	**Estimates**	***P*** **-value**	**Estimates**	***P*** **-value**
(Intercept)	8.09 (6.84–9.34)	**<0.001**	11.58 (10.24–12.91)	**<0.001**	−0.03 (−0.56 to 0.50)	0.91	61.65 (44.98–78.33)	**<0.001**	69.47 (59.63–79.32)	**<0.001**
Phase (2)	0.08 (−0.04 to 0.21)	0.17	0.01 (−0.13 to 0.14)	0.90	0.03 (−0.03 to 0.09)	0.35	−3.00 (−4.57 to −1.43)	**<0.001**	0.73 (−0.23 to 1.69)	0.14
Adopted (yes)	0.45 (0.21–0.70)	**<0.001**	0.57 (0.31–0.82)	**<0.001**	−0.01 (−0.10 to 0.08)	0.88	2.77 (−0.57 to 6.12)	0.10	0.15 (−1.61 to 2.18)	0.88
Age (years)	0.03 (−0.04 to 0.09)	0.44	−0.08 (−0.14 to −0.01)	**0.02**	−0.03 (−0.05 to −0.01)	**0.009**	−1.92 (−2.82 to −1.02)	**<0.001**	0.82 (0.31–1.32)	**0.002**
Gender (boy)	−0.09 (−0.28 to 0.08)	0.32	−0.07 (−0.25 to 0.12)	0.49	0.06 (−0.00 to 0.13)	0.05	−0.58 (−3.07 to 1.91)	0.65	−0.42 (−1.82 to 0.99)	0.56
Cosine peak	0.02 (−0.05 to 0.10)	0.55	−0.01 (−0.09 to 0.07)	0.76	0.05 (0.02–0.09)	**0.001**	−0.79 (−1.76 to 0.18)	0.11	0.26 (−0.32 to 0.85)	0.37
Mother's age	−0.01 (−0.03 to 0.01)	0.53	−0.02 (−0.04 to 0.00)	0.11	−0.01 (−0.01 to 0.00)	0.07	−0.11 (−0.38 to 0.15)	0.41	0.07 (−0.08 to 0.22)	0.36
Room-sharing with parents	−0.27 (−0.43 to −0.11)	**0.001**	−0.24 (−0.41 to −0.07)	**0.005**	−0.06 (−0.12 to 0.00)	0.06	1.54 (−0.63 to 3.70)	0.16	−0.75 (−2.01 to 0.51)	0.24
Spring	−0.02 (−0.19 to 0.14)	0.77	0.00 (−0.18 to 0.18)	0.10	0.02 (−0.05 to 0.10)	0.59	−0.50 (−2.69 to 1.69)	0.66	−0.20 (−1.52 to 1.12)	0.77
Summer	0.00 (−0.22 to 0.23)	0.97	0.26 (0.01–0.50)	**.04**	0.03 (−0.07 to 0.14)	0.53	2.28 (−0.65 to 5.22)	0.13	−2.00 (−3.79 to −0.22)	**0.03**
Winter	0.04 (−0.13 to 0.22)	0.62	0.17 (−0.02 to 0.35)	0.08	0.06 (−0.01 to 0.14)	0.10	0.94 (−1.32 to 3.20)	0.42	−0.80 (−2.17 to 0.56)	0.25
Phase ^*^ adoption status interaction	−0.22 (−0.40 to −0.05)	**0.01**	−0.34 (−0.53 to −0.15)	**<0.001**	−0.03 (−0.12 to 0.06)	0.53	−0.34 (−2.61 to 1.92)	0.77	0.39 (−1.00 to 1.78)	0.58
**Random effects**										
σ^2^ (variance of model)	0.11		0.14		0.03		18.51		7.04	
τ_00_ (variance between individuals)	0.21_id_		0.21_id_		0.02_id_		42.19_id_		12.75_id_	
ICC (intraclass correlation)	0.66		0.61		0.38		0.70		0.64	
N	145_id_		145_id_		145_id_		145_id_		145_id_	
Observations	271		271		271		271		271	
Marginal R^2^/conditional R^2^	0.103/0.691		0.204/0.693		0.143/0.466		0.256/0.773		0.113/0.691	

The bold values are the values with statistical significance.

The unconditional growth models for the sleep parameters (model with phase but without independent variables) showed changes (all *P*-values < 0.01) in the time spent in bed (intercept 11.37, slope −0.38), total sleep time (intercept 8.84, slope −0.21), sleep efficiency (intercept 78.0, slope 0.85), and the fragmentation index (intercept 39.0, slope −2.70) between the study phases. The covariances between the intercepts and slopes were negative in all but sleep efficiency measures, suggesting that higher baseline levels predicted steeper reductions in these variables.

The associations between adoption status and each of the sleep parameters are shown in Tables 2, 3. There were significant differences in total sleep time and time in bed between the controls and adopted children. These associations were robust to adjustments for age, gender, cosine peak, and mother's age. There were significant phase and adoption status interactions in the amount of time spent in bed and actual sleep time (Table 2), suggesting that there were also differences in developmental trajectories (i.e., a progressive increase or decrease) in the variables between the controls and adoptees during the study phases. Similar results were found in the nocturnal sleep indicators (Table 3). Both time in bed and total sleep time were longer in the adopted children, and the reductions in these variables were steeper among the adopted children during the follow-up. Figure 1 shows the mean trajectories for sleep duration and Figure 2 for time spent in bed in both groups over the study phases. In the second phase, held a year later, there were reductions in the differences in hours in bed and sleep between the adoptees and controls (Figures 1, 2; Tables 2, 3).

## Discussion

Our findings suggest that internationally adopted children did not sleep more poorly and did not experience substantially more sleep problems than their biological controls at preschool age when sleep was objectively recorded using actigraphy in the mean 0.8 years after arrival in their new family.

In our study, the adopted children slept more than their controls in both study recordings after age adjustment. During the 1-year follow-up, their sleep approached that of the controls. Even though internationally adopted children were younger than the controls and their sleep was more fragmented at the first assessment, our findings however suggest, that they slept more than the controls. The larger fragmentation of sleep of the internationally adopted children, which disappeared after age adjustment in the model, suggests that this was a logical finding as the adopted children were a year younger. Most of the adopted children were in home care with their other parent staying at home in Phase 1, which may explain their slightly later cosine peak, while the controls were in day care and used to daily routines.

Adopted children, especially international adoptees, can be considered a risk population for sleep problems due to their early life adversities. Internationally adopted children generally face many major psychosocial stressors during pregnancy and the early years (6, 15). They might face multiple traumatic events and acute and chronic stress prior to adoption, during transition, and after adoption. Long-lasting and recurrent trauma expositions can be considered a risk factor for sleep disturbances altering the neural circuits associated with regulatory skills (35–37). However, we know little about sleep difficulties among infants and preschool children with multiple and repeated traumas (38). The greater need for sleep among the internationally adopted children in our results indicates the need of further research into the potential compensatory mechanisms of developing brains.

Nevertheless, due to a lack of information about children's exact pre-adoptive experiences, we had no knowledge of the range of traumatic events which they were exposed to. Different types of maltreatment may have differential impacts on a child's sleep and self-regulation (39). Against this backdrop, it is not surprising that, in subjective studies, adoptive parents often report sleep disorders in their children (8–10). However, the current study found no significant objective differences in sleep problems between adopted children and biological controls. One explanatory factor may be that in our study, the proportion of adopted children with special needs (global developmental delay or disability) was quite small (Table 1). Sleep problems among children with special needs are more frequent than in normally developing children (40, 41).

Adversities in early life among internationally adopted children may lead to an increased number of attachment problems in comparison with their biological offspring (42). According to the attachment theory different attachment styles are formed between child and caregiver as a result of the experiences of nurture and affection. These models activate especially in times of need or as healthy or unhealthy coping strategies (43, 44). Later studies of attachment style and sleep suggest that there may be a relationship between lower attachment security and poorer sleep. However, the studies using actigraphy have not found association between actigraphic results and attachment (45). Also, in our study the larger sleep fragmentation among the adopted children in the first recording was explained with age which is in line with these studies.

Because of the living conditions in pre-adoptive orphanages, adopted children may face neglect and psychological deprivation. Sometimes, however, children in strict institutional settings might have learned exact bedtime routines. Furthermore, upon arrival in a new home country and family, internationally adopted children usually have growth and developmental delays (46–50), and during the first months in a new home country, they often demonstrate rapid catch-up processes in both growth and development (46, 47, 51). The pre-adoptive deprivation, the emerging new attachment relationship, and the catch-up processes may account for the larger need for sleep and longer stays in bed among internationally adopted children.

Our results also raise the question of why the adoptive parents may perceive their children to have sleep problems as reported in many questionnaire studies. This is no wonder considering above mentioned studies on attachment styles and sleep. In the review study Adams et al. also found differences between parental reports and actigraphic sleep pattern results of children (45). Prior to adoption, adoptive parents also undergo comprehensive training and are told about various challenges they might face with their newly adopted child. One reason may be as in our study that the adoptive parents were significantly older than the control parents, and often highly educated and have been in working life for some time. With a newly adopted child, they must rapidly adapt to different sleep patterns and everyday life with a child. On the other hand, similar phenomenon is seen in biological parents, even though they get used to the child's varied and changing sleep rhythms as the child grows, parents who themselves have sleep problems perceive and report more sleep problems among their biological children (19).

Family sleeping arrangements may also influence parents' perception of their children's sleep problems. In Phase 1 of our study, half the adopted children slept in the same room as their parents (Table 1). Although co-sleeping with parents can be a result of child sleep disturbances, in a study on toddler sleep problems, co-sleeping decreased maternal sleep (52). Another study about the sleep problems of internationally adopted children from China showed that children who shared a bed or room with their parents had higher sleep problem scores on the 7-item Sleep Problem Scale of the CBCL (Child Behavior Checklist) than those who slept in their own rooms (7). However, in that particular study, the sleep problem scores of the adopted children did not differ significantly from those of the normative CBCL sample (7).

Sleep rhythm and sleep patterns are learned and developed at a fairly young age (1, 5). Following this normal, biological development of self-regulatory skills, a child has ability to self-soothe at night and does not need parental help to soothe back to sleep. Most of the adopted children in our study moved to their adoptive family above the age of two, and some of them may have already learned their own bedtime routines and self-regulatory and self-soothing methods to fall asleep independently under orphanage circumstances. Also, they might be accustomed to staying in the bed longer as in many orphanages children are kept in bed for long periods of time compared with family customs. These notions could explain why the adopted children, contrary to our presuppositions, did not have significantly more sleep problems than the controls. Adopted children might also have more sleep problems soon after they arrive in the new family, as shown in a questionnaire-based study by Schenkels et al. where children had arrived in the new home < 6 months earlier (10). In their study, the adopted children were also 7 months younger than in our study. Recognizing this baseline, our finding involving somewhat older children who had stayed 3–4 months longer in their family suggests that the sleep of the adopted children quickly normalizes to levels that do not differ significantly from those of the controls.

Although internationally adopted children face many psychosocial stress factors in their early life, the adoption gives them a new opportunity. Post-adoption protective factors, such as a secure living environment and necessary loving care, might have influenced the positive sleep patterns of these children.

## Limitations

This study was unable to determine the exact sleep structure of these children because actigraphy cannot report brain electrical function. However, the study sought to understand how adopted children sleep, not describe their sleep structure. Though our results showed that the internationally adopted children do not sleep any worse than the control children in early years in their new families, it cannot not predict later life events such as school start or beginning of puberty. Further research with objective methods into how the adopted children sleep later in life, is still needed.

Even though actigraphy is a reliable method to evaluate children's sleep, the amount of total sleep time in actigraphy is usually shorter than in estimated by the parents in parental surveys or in the recommendations given to the population. Normal sleep involves small waking ups from deep sleep throughout the night, which may not be noticed by either the person themselves or the child's parents, but actigraphy recognizes these all (28, 53).

Although the analyses were adjusted for a wide range of potential confounders, there may have been unobserved confounding factors. Also, there might have been some individuals with poor sleep that did not appear to be remarkable at the group level. Children's experiences of trauma are known to be related to sleep problems, but in this study, we did not know the pre-adoption experiences and potential trauma of the study participants.

## Conclusions

Our results suggest that even though earlier studies based on parental reports of children's sleep showed that internationally adopted children have sleep problems, an objective assessment using actigraphy showed that they slept for longer periods and their sleep was not poorer than in biological controls. These encouraging results can ease the stress of adoptive parents with the knowledge that although the sleep of adopted children is different from that of parents, this does not necessarily translate into abnormality in their children's sleep.

## Data availability statement

The datasets presented in this article are not readily available because this article is based on health data and the data cannot be shared publicly because of GDPR, local protection act. Access to these data is regulated by Finnish legislation and FinData, and the Health and Social Data Permit Authority. The disclosure of data to third parties without explicit permission from Findata is prohibited. Only those fulfilling the requirements established by Finnish legislation and Findata for viewing confidential data are able to access the data. Requests to access the datasets should be directed to https://www.findata.fi/en/about-us/data-protection-and-the-processing-of-personal-data/.

## Ethics statement

The studies involving human participants were reviewed and approved by the Ethics Review Committee of the Hospital District of Southwest Finland. Written informed consent to participate in this study was provided by the participants' legal guardian/next of kin.

## Author contributions

A-RH, HL, IV, HRö, HRa, and ME conceptualized, designed the study, reviewed, and revised the manuscript. A-RH, HL, and HRö coordinated and collected the data. HL administrated the project, supervised the data collection, designed the data collection instruments, and obtained funding. A-RH drafted the initial manuscript and obtained funding. IV analyzed the original actigraphy data. ME analyzed the final data and supervised the drafting of the initial manuscript. All authors contributed to the manuscript and approved the final submitted version.

## Funding

HL received support for all the FinAdo Study phases from the Foundation for Pediatric Research, Finland, EVO (State Research Funding for University-Level Health Research) Grant from Turku University Hospital, and funding from the Signe and Ane Gyllenberg Foundation. A-RH received personal grants from the Foundation for Pediatric Research, Finland, the Arvo and Lea Ylppö Foundation, and the Finnish Brain Foundation for this work. ME was funded by the Academy of Finland (Grant Numbers: 265977 and 329224). HRö was supported by EVO Grant of the Turku University Hospital and a grant from the Foundation of Turku University Central Hospital. The funders had no role in the design and conduct of the study.

## Conflict of interest

The authors declare that the research was conducted in the absence of any commercial or financial relationships that could be construed as a potential conflict of interest.

## Publisher's note

All claims expressed in this article are solely those of the authors and do not necessarily represent those of their affiliated organizations, or those of the publisher, the editors and the reviewers. Any product that may be evaluated in this article, or claim that may be made by its manufacturer, is not guaranteed or endorsed by the publisher.
